# Two New Cases of *KIR3DP1*, *KIR2DL4*-Negative Genotypes, One of which is also Lacking *KIR3DL2*

**DOI:** 10.1007/s00005-014-0299-5

**Published:** 2014-07-18

**Authors:** Wanda Niepiekło-Miniewska, Natalia Żuk, Joanna Dubis, Maciej Kurpisz, David Senitzer, Anna Havrylyuk, Ryszard Grendziak, Wojciech Witkiewicz, Valentyna Chopyak, Piotr Kuśnierczyk

**Affiliations:** 1 Laboratory of Immunogenetics and Tissue Immunology, Department of Clinical Immunology, Ludwik Hirszfeld Institute of Immunology and Experimental Therapy, Polish Academy of Sciences, Rudolfa Weigla 12, 53-114 Wrocław, Poland; 2 Research and Development Centre, Regional Specialised Hospital, Wrocław, Poland; 3 Department of Reproductive Biology and Stem Cells, Institute of Human Genetics, Polish Academy of Sciences, Poznań, Poland; 4 City of Hope Comprehensive Cancer Center, Duarte, CA USA; 5 Department of Clinical Immunology and Allergology, Danylo Halytsky Lviv National Medical University, Lviv, Ukraine; 6 Department of Vascular Surgery, Regional Specialised Hospital, Wrocław, Poland

**Keywords:** Killer cell immunoglobulin-like receptor, Framework genes, Deletion

## Abstract

The killer immunoglobulin-like receptor (*KIR*) genes *KIR2DL4*, *KIR3DL2,* and *KIR3DP1* are present in virtually all humans. *KIR2DL4* encodes a receptor present on uterine and decidual natural killer (NK) cells and some peripheral blood NK cells. Its only known ligand is the human leukocyte antigen-G molecule expressed on extravillous trophoblasts, and on tissues in some diseases. KIR3DL2 binds HLA-A*03 and HLA-A*11 as well as HLA-B*27 dimers, and microbial CpG DNA. KIR3DP1 is a pseudogene. During our immunogenetic studies we found two individuals, one from Lower Silesia district in Poland, and another from Western Ukraine, who were reproducibly negative for *KIR2DL4* and *KIR3DP1* genes, using three different PCR systems. Both individuals displayed very similar genotypes, possessing only *KIR3DL3*, *KIR2DL3*, *KIR2DP1*, *KIR2DS1*, and probably a rare variant of *KIR2DL1*. The Pole had also *KIR3DL2*, which the Ukrainian was apparently lacking. The Lower Silesia has been populated after the Second World War by a remarkable percentage with displaced people from Western Ukraine, which might contribute to genetic similarity of the two individuals described here.

## Introduction

Killer cell immunoglobulin-like receptors (KIRs) are present on natural killer (NK) cells and some subpopulations of T lymphocytes. They have either inhibitory (long cytoplasmic tail-receptors KIR2DL and KIR3DL possessing two or three extracellular immunoglobulin-like domains, respectively) or activating (short cytoplasmic tail-receptors KIR2DS or KIR3DS) properties upon binding a ligand. Genes for majority of KIRs are not present in all individuals but only in a fraction of them (haplotypic polymorphism). However, some, so called framework genes, namely *KIR3DL3*, *KIR3DP1*, *KIR2DL4,* and *KIR3DL2*, are present in virtually all genotypes (Jiang et al. 2012). Among these, *KIR2DL4* gene has special properties: it codes for structural features characteristic for both inhibitory and activating KIR; it is expressed in all NK cells whereas other KIRs are rather distributed clonally on some NK cells; and in contrast to other KIRs which function as cell surface receptors, it is localized, in resting NK cells, in endosomes where it may bind its ligand, a soluble HLA-G molecule (Rajagopalan and Long 2012), although this view has been recently challenged (Le Page et al. 2014). KIR2DL4 molecule appears as a cell surface receptor on uterine and decidual NK cells and some (mostly activated) peripheral blood NK cells (Goodridge et al. 2009; Hromadnikova et al. 2013; Yan et al. 2007). KIR3DL2 binds HLA-A*03 and HLA-A*11 as well as HLA-B*27 dimers, and also a microbial CpG DNA (Shaw and Kollnberger 2012; Sivori et al. 2010). KIR3DP1 is a pseudogene (Jiang et al. 2012). Here, we describe two cases of *KIR2DL4*-negative individuals detected in the Polish and Ukrainian populations. Both individuals present similar deletion of not only *KIR2DL4* but also neighboring *KIR* genes, including *KIR3DP1*. Interestingly, the Ukrainian individual was also lacking another framework gene, *KIR3DL2*.

## Materials and Methods

### Study Subjects

The study was approved by the Local Ethical Committee at the Research and Development Centre, Regional Specialised Hospital, Wrocław, the Bioethics Committee of the Medical University of Poznań, and the Bioethics Committee of the Danylo Halytsky Lviv National Medical University.

*No. 89* a 69-year-old Polish male, 82 kg body weight, 171 cm high, body mass index (BMI) of 28.04, with arterial hypertension, morbus ischemicus cordis and aspirin idiosyncrazy but otherwise healthy and without genetic diseases in family.

*No. 175* *K* a 67-year-old Polish male, 90 kg body weight, 177 cm high, BMI of 28.73, with total cholesterol and glucose at upper border of the norm, atherosclerosis, polycystic kidney disease, varices, nosebleed, and osteoporosis.

*No. 24* a 22-year-old healthy male from western Ukraine, HIV-, HBV- and HCV-negative blood donor, recruited to a control group for our study on cryptorchidism (Kurpisz et al. 2011).

*No. 58* a 19-year-old healthy male from western Ukraine, HIV-, HBV- and HCV-negative blood donor, recruited to a control group for our study on cryptorchidism (Kurpisz et al. 2011).

### DNA Isolation

Genomic DNA was isolated from venous blood using QIAamp DNA Blood Mini Kit Qiagen (Qiagen GmbH, Hilden, Germany), according to manufacturer’s recommendations. DNA concentration was measured in NanoDrop 2000 UV–Vis Spectrophotometer (Thermo Scientific, NanoDrop Products, Wilmington, Delaware, USA).

### KIR Typing

Three PCR-sequence-specific primers (SSP) systems were used

- Olerup SSP KIR Genotyping kit (Olerup GmbH, Vienna, Austria), lot No. 07 N including *Taq* polymerase. PCR-SSP procedure was performed according to manufacturer’s instruction.
- SSP as described by Vilches et al. (2007) that enable to amplify short DNA fragments (96–158 bp), in our modification (Niepieklo-Miniewska et al. 2013)
- Multiplex PCR-SSP (four PCR reactions containing primers for 3–4 *KIR* genes each) as published earlier (Nowak et al. 2011; Sun et al. 2004).

Our *KIR* typing has been validated three times per year by the International KIR Exchange program organized by the Immunogenetics Center of the University of California at Los Angeles.

## Results

**Fig. 1:**
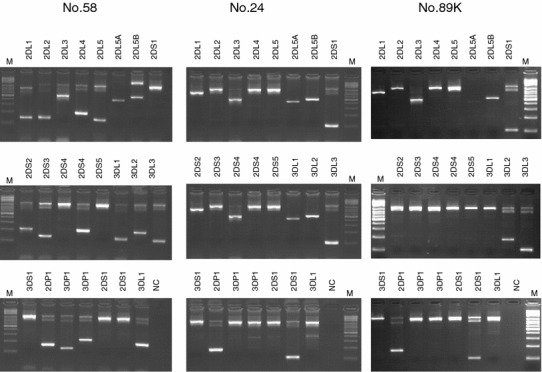
Results of *KIR* typing using Olerup KIR Genotyping kit™. Interpretation of all results is shown in Table 1 and described in the text. M: marker of the molecular mass; NC: negative control. Polish individual No. 89 K; Ukrainian individual No. 24; Ukrainian individual No. 58

**Fig. 2:**
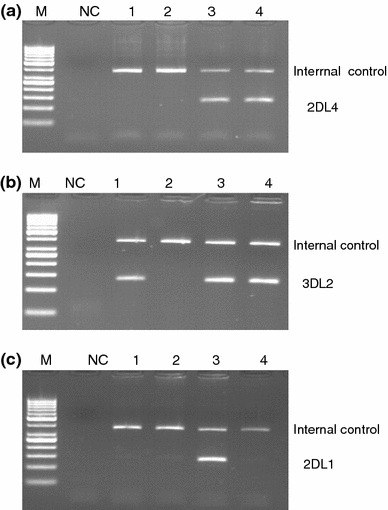
Results of *KIR* typing using primers and PCR conditions described by Vilches et al. (2007). **a** *KIR2DL4* typing of samples No. 89 K, No. 24 and two unrelated *KIR2DL4*-positive individuals. Both No. 89 K (*lane 1)* and No. 24 (*lane 2*) were negative (only internal control gave a product), whereas both positive control samples (*lanes 3* and *4*) gave *KIR2DL4* amplicons. **b** *KIR3DL2* typing of the same samples. Only sample No. 24 (*lane 2*) was negative for framework gene *KIR3DL2*, whereas both sample No. 89 K (*lane 1*) and unrelated positive control samples (*lanes 3* and *4*) gave positive reactions. **c** *KIR2DL1* typing of the same samples. Only control sample shown in *lane 3* was positive, whereas all other samples gave no product

**Fig. 3:**
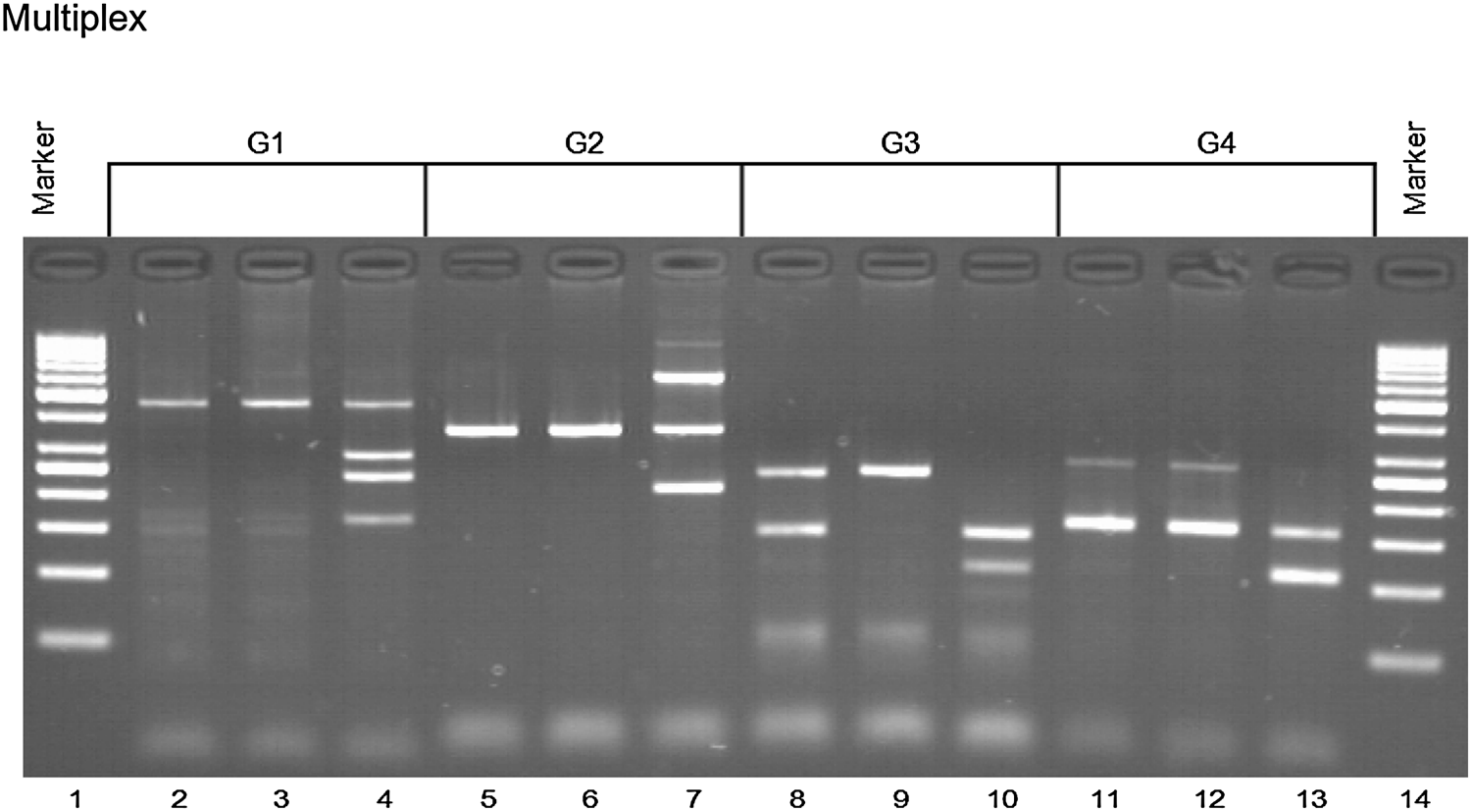
Results of KIR typing using multiplex PCR-SSP kit (Sun et al. 2004). Samples No. 89 K (*lanes 2*, *5*, *8* and *11*), No. 24 (*lanes 3*, *6*, *9* and *12*) and framework gene-positive control sample No. 175 K (*lanes 4*, *7*, *10* and *13*) were tested for the presence of particular KIR gene groups as shown, together with results, in Table 2. Please note the absence of KIR2DL4 (G1) in samples No. 89 K and 24, and absence of KIR3DL2 (G3) in sample No. 24. KIR2DL1 (G1) gave clearly positive band in samples No. 89 K and 24 in this test, in contrast to negative results of these samples with both Olerup (Fig. 1) and Vilches et al. (2007) (Fig. 2) primers. Summary of these results is given in Table 2

Upon genomic *KIR* typing using commercial Olerup kit (see Materials and Methods) in disease association studies on Polish and Ukrainian patients and respective control groups, we noticed a lack of *KIR2DL4* gene in one Polish (No. 89 K) and one Ukrainian (No. 24) control individual (Fig. 1). All other samples from both control and patient groups were positive (Fig. 1). The individual No. 24 apparently was lacking another framework gene, *KIR3DL2*, whereas another Ukrainian control individual, No. 58, possessed both genes (Fig. 1). To confirm this result, we repeated *KIR* typing using Olerup kit once more, with the same result (data not shown, and see Table 1). Furthermore, we checked the presence of *KIR2DL4* and *KIR3DL2* genes in these samples using primers published by Vilches et al. (2007) (Fig. 2). Finally, we typed these samples again with the use of multiplex PCR-SSP described previously (Sun et al. 2004) (Fig. 3). Our individuals No. 89 K and No. 24 were reproducibly *KIR2DL4*-negative in all these tests, in contrast to other individuals who were all positive (Fig. 1, 2, 3, and results not shown). They also lacked *KIR3DP1* which is present whenever *KIR2DL4* is present, with extremely rare exceptions (Jiang et al. 2012). They possessed *KIR3DL3*, *2DL3*, *2DP1*, and *2DS1,* and No. 89 K had also *KIR3DL2*, absent from No. 24 genotype. Their genotypes contained *KIR2DL3* gene, characteristic for the centromeric segment of the KIR region, but another centromeric gene, *KIR2DL1*, gave inconsistent results: it was detected by multiplex test (Fig. 3; Table 2) but neither by commercial test (Fig. 1; Table 1) nor by PCR-SSP using primers described by Vilches et al. (2007) (Fig. 2).

**Table 1:** Summary of the results of testing individuals No. 89 K, No. 24 and No. 58 with Olerup PCR-SSP kit shown in Fig. 1 and second testing with identical results

Primer mix	Size of specific PCR product (bp)	Size of control band (bp)	KIR gene	No. 89 K	No. 24	No. 58
1	145	800	2DL1	–	–	**+**
2	145	1070	2DL2	–	–	**+**
3	520	1070	2DL3	**+**	**+**	**+**
4	200	1070	**2DL4**	–	–	**+**
5	155	1070	2DL5A, 2DL5B	–	–	**+**
6	1650	**430**	2DL5A	–	–	–
7	1650	**515**	2DL5B	–	–	**+**
8	100	1070	2DS1	**+**	**+**	–
9	205	1070	2DS2	–	–	**+**
10	160	1070	2DS3	–	–	**+**
11	215	1070	2DS4	–	–	–
12	200	1070	2DS4	–	–	**+**
13	110	1070	2DS5	–	–	–
14	130	1070	3DL1	–	–	**+**
15	95	1070	**3DL2**	**+**	–	**+**
16	115	1070	3DL3	**+**	**+**	**+**
17	130	1070	3DS1	–	–	–
18	165	1070	2DP1	**+**	**+**	**+**
19	125	1070	**3DP1**	–	–	**+**
20	235	1070	3DP1	–	–	**+**
21	145	1070	2DS1*001	–	–	–
22	95	1070	2DS1*002-008	**+**	**+**	–
23	80	1070	3DL1*00401-00403, 019, 021, 036, 037, 039, 056, 072	–	–	**+**
24	–	–	**Negative control**			

Framework genes not detected in described individuals are show in bold

**Table 2:** Interpretation of results of multiplex PCR-SSP KIR typing shown in Fig. 3

Multiplex group	KIR gene	Amplicon (bp)	89 K	24	175 K
1	2DL1	437	+	+	+
	2DS3	279	–	–	+
	**2DL4**	**232**	–	–	**+**
	2DL2	164	–	–	+
2	3DL1	565	–	–	+
	2DL3	334	+	+	+
	2DS2	204	–	–	+
	3DS1	171	–	–	–
3	2DS1	231	+	+	–
	**3DL2**	**142**	**+**	–	**+**
	2DL5	113	–	–	+
4	2DS5	194	–	–	–
	3DL3	155	+	+	+
	2DS4n	130	–	–	–
	2DS4d	108	–	–	+

Framework genes not detected in described individuals are show in bold

## Discussion

**Fig. 4:**
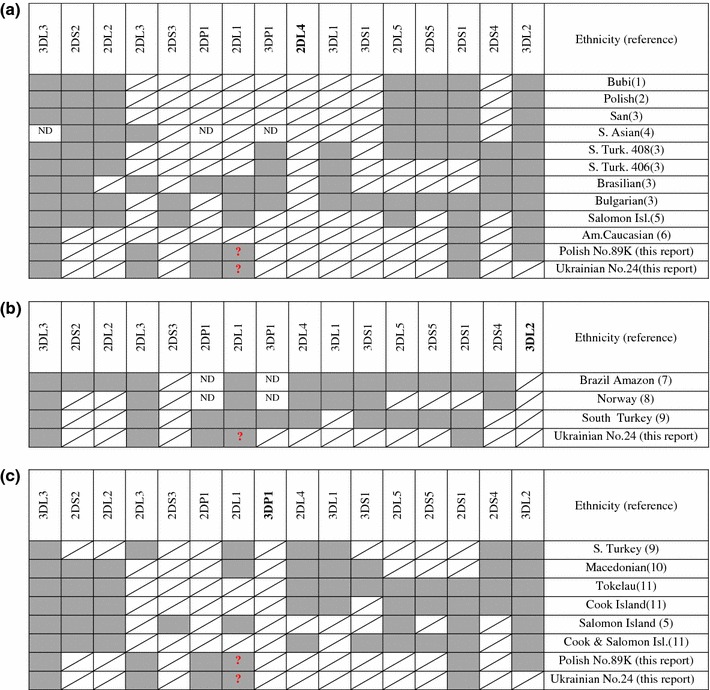
*KIR* genotypes of published framework *KIR*-negative individuals. **a** *KIR2DL4*-negative individuals. *Gray shadowed cells* gene present, *white diagonally crossed cells* gene absent, *question mark* gene presence dubious (inconsistent results), *ND* not done. **b** KIR genotypes of published *KIR3DL2*-negative individuals. Designations of cells as in Fig. 4a. **c** *KIR* genotypes of published *KIR3DP1*-negative individuals. Designations of cells as in Fig. 4a. References: *1* Gomez-Lozano et al. 2003, *2* Nowak et al. 2011, *3* Gonzalez-Galarza et al. 2011, *4* Norman et al. 2002, *5* Taniguchi and Kawabata 2009, *6* Traherne et al. 2010, *7* Ewerton et al. 2007, *8* Karlsen et al. 2007 *9* Ozturk et al. 2012 *10* Djulejic et al. 2010, *11* Velickovic et al. 2006

We have detected two *KIR3DP1*, *KIR2DL4*-negative individuals, one of which was also negative for the third framework gene, *KIR3DL2*. Such genotypes are extremely rare in world populations (Djulejic et al. 2010; Ewerton et al. 2007; Gomez-Lozano et al. 2003; Gonzalez-Galarza et al. 2011; Karlsen et al. 2007; Norman et al. 2002; Nowak et al. 2011; Ozturk et al. 2012; Taniguchi and Kawabata 2009; Traherne et al. 2010; Velickovic et al. 2006), and even haplotypes contributing to them, most frequently in heterozygotic configuration, are very rare (Gonzalez-Galarza et al. 2011; Jiang et al. 2012; Pyo et al. 2010; 2013; Traherne et al. 2010). We found in the www.allelefrequencies.net database (Gonzalez-Galarza et al. 2011) only ten *KIR2DL4*-negative genotypes in addition to two new ones described here (Fig. 4a), three *KIR3DL2*-negative genotypes plus our Ukrainian (Fig. 4b), and six *KIR3DP1*-negative genotypes plus two described here (Fig. 4c) out of nearly 13,000 individuals tested worldwide (Gonzalez-Galarza et al. 2011). This speaks in favor of a strong positive selection for all three genes, including *KIR3DP1*. This pseudogene might be suspected to be preserved simply due to its close proximity to *KIR2DL4* which is likely strongly selected for. However, *KIR3DP1* is located on centromeric segment of KIR locus, whereas *KIR2DL4* is on telomeric segment (Jiang et al. 2012; Pyo et al. 2013) which seems to exclude the close localization as a reason for preservation of *KIR3DP1* together with *KIR2DL4*. Nevertheless, genotypes containing *KIR2DL4,* but not *KIR3DP1,* are extremely rare, as only few such genotypes from Cook, Samoa, Tokelau, and Tonga Islands as well as from Turkey have been described so far (Velickovic et al. 2006) (Fig. 4c), and opposite configuration, *KIR3DP1* ^+^, *KIR2DL4* ^–^, was found in only single individuals from South Turkey, Brazil, and Bulgaria (Fig. 4a). Homozygous *KIR3DL2* deletion is also very infrequent, as only three such cases exist in the database (Gonzalez-Galarza et al. 2011) and our Ukrainian No. 24 is the fourth one (Fig. 4b). Haplotypes with this deletion, but not in homozygous configuration, have been identified in some individuals by gene copy number detection (Pyo et al. 2013). The KIR3DL2 molecule plays an important role in both innate and adaptive immunity. It is expressed on NK cells, as well as on subpopulation(s) of T lymphocytes (Bowness et al. 2011; Wong-Baeza et al. 2013). It binds not only HLA-A*03 and HLA-A*11 heterotrimers as well as HLA-B*27 homodimers, but also microbial CpG DNA (Shaw and Kollnberger 2012; Sivori et al. 2010). It has the highest number of alleles, except for *KIR3DL1/S1*. This is suggestive of a strong selection for polymorphism (Shaw and Kollnberger 2012).

In some very rare American haplotypes, most of the *KIR3DL*2 gene (intron 3 to beyond exon 9) was deleted, giving a typing pattern identical to our No. 24. Such a haplotype has also been detected in an American of Ukrainian descent, in addition in homozygous configuration (Traherne et al. 2010).

Interestingly, Lower Silesia was populated in remarkable fraction, after the Second World War, by people resettled from Western Ukraine. Therefore, not only No. 24 but also No. 89 K genotype may be derived from there, whence similarity between these genotypes and with haplotypes described in the USA and mentioned above. It has been established by sequencing that all American *KIR3DL2* deletion haplotypes contained exons 1–3 from KIR3DL2*007 allele, suggesting a recent common origin. It would be desirable then to check whether our genotypes No. 89 K and No. 24 bear the same allele that may be derived from the same recombination event.

We had a problem with establishing whether *KIR2DL1* gene is present in our genotypes No. 89 K and No. 24. The reagents from both Olerup and Vilches et al. (2007) PCR-SSP gave reproducibly negative results, whereas in the multiplex assay both individuals were clearly positive (Figs. 1, 2, 3; Tables 1, 2). It is likely that both our individuals possess genotypes similar to the haplotypes described earlier in Northern American Caucasian population (Pyo et al. 2013; Traherne et al. 2010) which contained a *KIR2DL1/KIR2DS1* hybrid resulting from recombination event which deleted all *KIR* genes located between these two genes. This may explain why some PCRs detected *KIR2DL1* in our samples, but other did not.

In conclusion, we found in our Slavic populations (Polish and Ukrainian), two individuals lacking several framework *KIR* genes, *KIR3DP1,* and *KIR2DL4*, and apparently also *KIR2DL1*. One of them was also negative for *KIR3DL2*. Both these individuals were relatively healthy in spite of very limited *KIR* gene repertoire. However, such genotypes are extremely rare in world populations. Therefore, studies on much larger populations would be required to determine the effect(s) of these deletions on the health of such individuals.
